# Trends in Physical Fitness Among School-Aged Children and Adolescents: A Systematic Review

**DOI:** 10.3389/fped.2020.627529

**Published:** 2020-12-11

**Authors:** Bojan Masanovic, Jovan Gardasevic, Adilson Marques, Miguel Peralta, Yolanda Demetriou, David Joseph Sturm, Stevo Popovic

**Affiliations:** ^1^Faculty for Sport and Physical Education, University of Montenegro, Niksic, Montenegro; ^2^Montenegrosport, Podgorica, Montenegro; ^3^Montenegrin Sports Academy, Podgorica, Montenegro; ^4^Faculdade de Motricidade Humana, Centro Interdisciplinar para o Estudo da Performance Humana, Universidade de Lisboa, Lisboa, Portugal; ^5^Faculdade de Medicina, Instituto de Saúde Ambiental, Universidade de Lisboa, Lisboa, Portugal; ^6^Department of Sport and Health Sciences, Technical University of Munich, Munich, Germany

**Keywords:** secular trends, physical fitness, fitness changes, physical performance, youngsters

## Abstract

**Introduction and Objective:** This systematic review aimed to analyse the international evolution of fitness with its distributional changes in the performance on tests of physical fitness among school-aged children and adolescents.

**Methods:** In accordance with the Preferred Reporting Items for Systematic Reviews and Meta-Analyses (PRISMA) guidelines, the search was undertaken in four international databases (ERIC, PubMed, Scopus, and Web of Science) to identify the studies reporting temporal trends in the physical fitness among school-aged children and adolescents.

**Results:** A total of 485 potential articles were identified, of which 19 articles were relevant for the qualitative synthesis; 1,746,023 children and adolescents from 14 countries (China, Finland, Sweden, Belgium, New Zealand, Denmark, Spain, Norway, Mozambique, Poland, USA, Lithuania, Portugal, Canada), for the period between 1969 and 2017 were included. The subjects were tested using 45 motor tests from eight battery tests. The quality of the study in eight articles was rated as strong, while in 11 articles it was rated as moderate.

**Discussion:** The vast majority of studies show a constant decline in strength and endurance. Three Chinese studies show an increase in strength from 1985 to 1995 and then a decline until 2014. For endurance, similar patterns were found in the two most comprehensive Chinese studies. The decline in flexibility is also evident in European countries. For agility, speed, balance, and coordination, the trend differs among populations.

## Introduction

Physical fitness is a multicomponent construct that is closely related to the ability to perform physical activity (1, 2). It is considered to be an important health marker, because high levels of fitness during childhood and adolescence have a positive impact on adult health (3, 4). Additionally, higher levels of physical fitness enable participation in a variety of physical activities and decrease the risk of health problems (5–7).

Physical fitness is determined by genetic factors and the level of regular exercise and physical activity (8). The modern era has brought changes in ways of life and work that are associated with lower levels of physical activity (9). From a traditionally active lifestyle in which physical fitness was necessary to manage daily tasks, most people switched to the more sedentary lifestyles. In the previous four decades, studies have indicated an association between the lower physical activity levels (10) and change in body composition and somatotype (11, 12). Also, declines in physical fitness are often recorded (13), which are likely influenced by the decreasing trend in physical activity and changes in body composition (14, 15). The decline in cardiorespiratory fitness has been extensively documented (16–18). Moreover, a decline in flexibility (19), repetitive strength and running speed were also recorded (20). Given the reported declining trends in physical activity and consequently physical fitness, some researchers predict the emergence of serious public health concerns (6, 21). Contrary to this evidence, in some countries, an increase has been registered on certain components of physical fitness. Examples of this include an increase of muscular fitness in Finland (22) and cardiorespiratory fitness and strength in Canada (23).

These differences in physical fitness among countries have not been examined in detail to date (3). Furthermore, previous studies have only analyzed trends of single physical fitness components (e.g., cardiorespiratory fitness, muscular fitness), which underscores the need for a comprehensive review study that covers more than one physical fitness component and gives a general picture of its trends, which would facilitate conclusion drawing and monitoring. Thus, the objective of this systematic review was to gather the available information on all components of physical fitness among children and adolescents and to analyse the international trends of their performances on the respective physical fitness tests.

## Methods

Data selection, collection, and analyses were performed in accordance with the Preferred Reporting Items for Systematic Reviews and Meta-Analyses (PRISMA) guidelines (24).

### Inclusion Criteria

Scientific articles containing data on temporal trends in physical fitness published up to April 2020 were included. Eligibility criteria were the following: (1) cross-sectional, longitudinal, and interventional studies (design criterion); (2) articles published in scientific journals, book chapters, books, conference proceedings, and theses (publish criterion); (3) fitness battery, physical fitness, cardiovascular fitness, speed, flexibility, agility, muscular strength, body composition (outcome measure criterion); (4) children and adolescents aged 10 to 18 years (participant criterion); (5) articles published in English, French, Portuguese, Spanish, or German (language criterion).

### Search Strategy

The literature search was undertaken in four international databases: the Education Resources Information Center (ERIC), PubMed, Scopus, and Web of Science. The search was conducted on April 17, 2020. In each database, a search was conducted by title, taking a predefined combination of keywords (through discussion among the research team) into account. The combination of used keywords was the following: “field-based test” OR fit^*^ OR “physical performance” OR “sport performance” OR “physical condition” OR “aerobic capacity” OR “maximum oxygen consumption” OR strength OR flexibility OR motor OR endurance OR speed OR agility OR balance OR “body composition” OR anthropometry OR “body mass index” OR BMI OR skinfolds OR “waist circumference” AND trend^*^ OR tendenc^*^ AND adolescent^*^ OR child^*^ OR young^*^ OR “school age” OR school-age OR youth. The keywords were selected through discussion among the research team, finally defined by the consensus of all authors.

### Data Extraction and Selection

After performing a search in the databases, the necessary data were transferred to a software tool for publishing and managing bibliographies. The process of data extraction was performed based on PRISMA guidelines (24). The articles were downloaded from the databases, after which duplicates, identified by title and author, were removed. Two researchers screened titles and abstracts of the remaining records. Then, the full-text of relevant articles were read and examined according to the inclusion criteria, to decide whether or not to include them in the systematic review. The following information was extracted from each study: author's name and year of publication, study design, country, sample characteristics (number of participants, gender, and age), the instrument/battery for assessing physical fitness, main results, and study quality.

### Study Quality and Risk of Bias

The methodological quality of the studies was assessed using the Quality Assessment Tool for Quantitative Studies (25), which is a 19-item checklist, assessing eight methodological domains: selection bias, study design, confounders, blinding, data collection methods, withdrawals and dropouts, intervention integrity, and analyses. Each section was graded as being of strong, moderate, or weak methodological quality. A global rating is determined based on the scores of each component. Two researchers rated the studies in each domain, as well as the overall quality of each study. Discrepancies were resolved by consensus.

## Results

**Figure 1:**
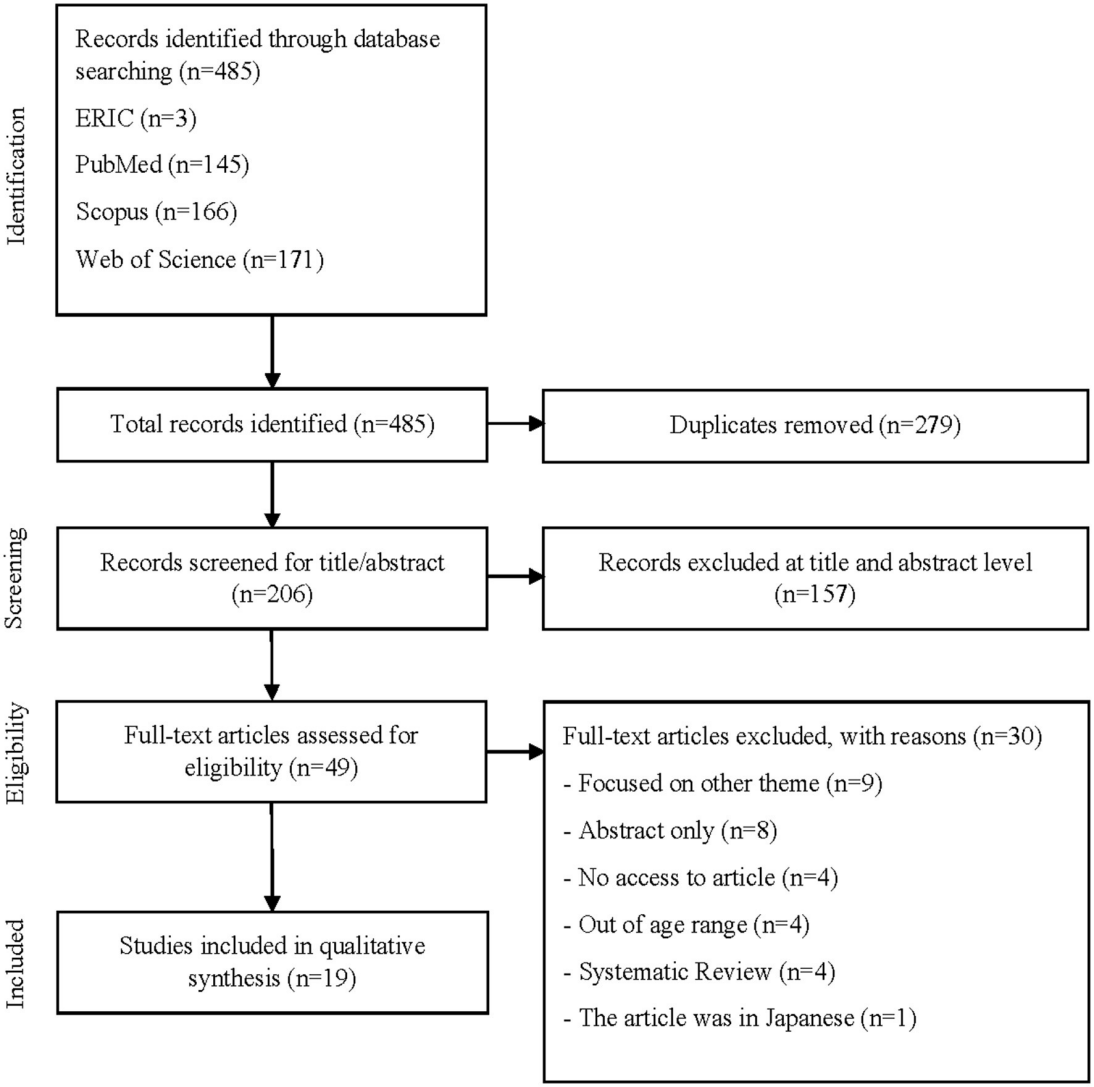
Flow diagram of study selection.

Figure 1 shows the flow chart of records selection. A total of 485 potential articles were identified through the electronic database search (three from ERIC; 145 from PubMed; 166 from Scopus; 171 from Web of Science). After exclusion of the duplicates (279), the title and abstract of 206 were assessed for eligibility. After elimination at the title and abstract level 157 articles, the remaining 49 articles were subsequently read. After reading, another 30 articles were eliminated, leaving 19 relevant articles that satisfied the inclusion criteria and were included in the qualitative synthesis.

### Study Characteristics

In Table 1, the studies' characteristics are presented. From 19 studies included in the qualitative synthesis, a total sample of 1,746,023 children and adolescents from 14 countries was represented. All studies had a cross-sectional design with two, three, or more samples from the same number of time-points. Three studies were performed in China and another three in Finland, two in Sweden, and one in each of the following countries: Belgium, New Zealand, Denmark, Spain, Norway, Mozambique, Poland, the United States of America, Lithuania, Portugal, and Canada. The period covered by the studies was between 1969 and 2017. Overall, participants' performance in 45 fitness tests from eight different fitness tests batteries was recorded. Strength was most frequently tested, followed by endurance, flexibility, agility, speed, balance, and coordination. The quality of the studies in eight articles was rated as strong, while in 11 articles it was rated as moderate.

**Table 1:** Characteristics of the studies.

**Author, Year**	**Study design**	**Country**	**Sample characteristics (number of participants, gender, age)**	**The instrument/battery for assessing physical fitness**	**Main results**	**Study quality**
Westerstahl et al. (26)	Cross-sectional	Sweden	*n* = 855: 417 girls, 426 boys; mean age 16.4	Sit-ups (number), Bench-press (number), Sargent jump (cm), Two-hand lift (N), Run-walk (m/9 min)	Both girls and boys performed less well in bench-press, sit-ups, and run-walk tests in 1995 compared to 1974. Boys performed better in the Sargent jump in 1995 than in 1974, but there was no difference among the girls for this test. Both girls and boys performed better in the two-hand lift in 1995 than in 1974. There were decreased aerobic fitness and an increased maximal static strength among adolescents in Sweden between 1974 and 1995	Strong
Matton et al. (27)	Cross-sectional	Belgium	*n* = 19,999: 11,899 boys (in 1969–1974), 4,899 girls (in 1979–1980), 1,429 boys (in 2005), 1,772 girls (in 2005) aged 12–18 years	Cross Sectional: Bent-arm hang (s), Sit-and-reach (cm), Flamingo balance (*n*/min), 10 × 5 m shuttle run (s); Parent offspring study: Plate tapping (*n*/20 s), Vertical jump (cm), Arm pull (kg), Leg lifts (*n*/20 s)	Boys tested in 2005 perform significantly better on the 10 × 5 m shuttle run test, but they perform significantly worse on sit-and-reach and bent-arm hang in cohort 12- to 14-year-olds compared to 1969–1974. Girls tested in 2005 perform significantly worse on bent-arm hang, flamingo balance, 10 × 5 m shuttle run test, and sit-and-reach for cohort 14 year-olds compared to 1969–1974. Boys (2002–2004) perform significantly worse on sit-and-reach plate tapping, and arm pulls (1969–1974). Girls (2002–2004) perform significantly worse on bent-arm hang, sit-and-reach, 10 × 5 m shuttle run, plate tapping, vertical jump, and arm pull compared to their mothers (1979–1980)	Moderate
Huotan et al. (14)	Cross-sectional	Finland	*n* = 1,275: 717 adolescents (384 boys, 333 girls) took part in the 1976 study, 558 (305 boys, 253 girls) took part in the 2001 study; aged 13–18 years	2,000 m running (s) for boys and a 1,500 m running (s) for girls	The mean 2,000 m running test time was longer in 2001 compared to 1976 in boys. The mean 1,500 m running test time was longer in 2001 compared to 1976 in girls, which indicates that on average, a boy in 2001 would finish about 180 m behind the average 1976 boy over the 2,000 m distance. In girls, the corresponding difference in the 1,500 m run was 83 m	Strong
Albon et al. (28)	Cross-sectional	New Zealand	*n* = 3,306: 1,456 girls, 1,850 boys aged 10–14 years	Sit-and-reach (cm), 4 × 9 m run (s), Abdominal curl ups (*n*), Standing broad jump (cm), 550 m run (s); AAHPER battery	Results on the 550-m run test decreased for boys (1.5%) and girls (1.7%), respectively. Results on the 4 × 9 m agility run test decreased for boys (0.2%) and girls (0.4%), respectively. Standing broad jump results for boys and girls in 2003 was less than their 1991 counterparts (0.3 and 0.2% per year for boys and girls, respectively). In contrast, for boys, performance in the curl-up test improvement is 1.5% per year, whereas improvement was of 2.1% per year for girls. Similarly, boys and girls were more flexible in 2003 compared to 1991; the improvement is 2.8 and 1.8% per year for boys and girls, respectively	Moderate
Andersen et al. (29)	Cross-sectional	Denmark	*n* = 1,050: 466 boys, 584 girls; aged 15–19 years	The VO2 max was estimated from maximal power output in a cycle test with the progressively increasing workload on a mechanically braked cycle ergometer (Monark 839, Varberg, Sweden)	No change in cardiorespiratory fitness over time (1983, 1997, 2003), and quite high levels were found in these representative cohorts. It founded substantial differences in maximal power output, but no differences when VO2 max was estimated from the equations derived in the validation studies	Moderate
Moliner-Urdiales et al. (3)	Cross-sectional	Spain	*n* = 731: 339 adolescents (155 boys, 184 girls) obtained between 2001 and 2002, 392 adolescents (206 boys, 186 girls) obtained between 2006 and 2007; aged 12.5–17.5 years	Handgrip strength (kg), bent-arm hang (s), Standing broad jump (cm), 4 × 10 m shuttle run (s), 20 m shuttle run (stages)	Performance in 4 × 10 m shuttle run and 20 m shuttle run tests was higher in 2006–2007 compared with 2001–2002. Performance in handgrip strength and standing broad jump tests was lower in 2006–2007 compared with 2001–2002. The bent-arm hang test was not significantly different. Levels of both speed/agility and cardiorespiratory fitness were higher in 2006–2007 than in 2001–2002. Upper body muscular strength is on the same level	Moderate
Huotari et al. (22)	Cross-sectional	Finland	*n* = 1,222: 643 adolescents (312 boys, 331 girls) took part in the 1976 study, 579 adolescents (308 boys, 271 girls) in the 2001 study; aged 13–16 years	Standing broad jump (cm), Sit-ups (*n*/30 s), 4 × 10 m shuttle run (s) for both sexes. Besides, Flexed-arm hang (n/s) was measured in girls and Pull-ups (repetition max) in boys	No statistically significant changes in the standing broad jump results among boys from 1976 to 2001, in girls a mild tendency toward higher scores (1.9%). Upper body muscular fitness in boys decreases by 21.2% (averaged pull-ups number had fallen). The results of the girls' flexed-arm hang test showed no difference. Sit-ups improved significantly over time in both sexes, in boys (13.6%), in girls (9.1%). The results for the agility 4 × 10 m shuttle run had also improved over time, 4.7% in boys, and by 2.3% in girls	Moderate
Dyrstad et al. (17)	Cross-sectional	Norway	*n* = 4,006 pupils: 2,384 boys, 1,622 girls; age 16–18	3,000 m running (s)	The 3,000 m running time decreases from the 1969 s to the 1979 s for boys. The distribution showed an increase in aerobic fitness in this decade. The running times have increased from the 1980 s to the 2000 s for boys and girls, respectively. The distribution showed a decline in aerobic fitness by 10 and 6%. The cohort of 16- to 18-year-old boys and girls in the decade 2000–2009 had a poorer aerobic fitness performance in the 3,000 m running test compared with earlier decades	Moderate
Dos Santos et al. (30)	Cross-sectional	Mozambique	*n* = 3,851: 591 subjects in 1992 (276 boys, 315 girls), 1,840 subjects in 1999 (854 boys, 986 girls), 1,420 subjects in 2012 (661 boys, 759 girls); aged 8–15 years	Handgrip strength (kg), 10 × 5 m shuttle run (s) from EUROFIT battery; Sit-and-reach (cm), 1-mile run-walk (s) from AAHPERD and FITNESSGRAM batteries, respectively	Children in 1992 were more flexible than those from 2012. Boys' handgrip strength increased from 1992 to 2012, while girls decreased their handgrip strength. Youth in 1992 were faster and more agile than their 2012 peers. A decrease was observed in cardiorespiratory fitness between 1992 and 1999 and between 1992 and 2012 for both sexes	Moderate
Karpowicz et al. (31)	Cross-sectional	Poland	*n* = 169: 21 girls (measured in the 2006), 21 (2007), 20 (2008), 21 (2009), 21 (2010), 21 (2011), 22 (2012), 22 (2013); mean age 15.5 ± 0.5	50 m sprint (s), 800 m endurance running (s), 4 × 10 m shuttle run (s), Standing long jump (cm), sit-ups (*n*/30 s), Handgrip strength (kg), Flexed-arm hang (*n*), Standing trunk flexion (cm); International Physical Fitness Test (IPFT) battery	The overall physical fitness of young women basketball players has been declining year by year. Girls obtained lower results in 6 of 8 tests (50 m run, Long jump, Flexed-arm hang, 4 × 10 m run, Sit-ups, Standing forward bend), compared with members of the Teams in 2006, with the differences for long jump, arms hang, sit-ups, and trunk flexion tests being statistically insignificant. A slight improvement was observed only in the 800 m run and hand strength	Moderate
Morales-Demori et al. (32)	Cross-sectional	USA	*n* = 435: 249 (male), 186 (female) healthy children and adolescents, aged 4–18 years, mean age 12.6 ± 3.2 years	Bruce protocol treadmill test	There was a significant difference in the mean endurance time between children grouped in 5-year intervals (1983–1990, 1991–1995, 1996–2000, 2001–2005, 2006–2010) with a significant downward trend in endurance time over the years, especially after 2001	Strong
Venckunas et al. (33)	Cross-sectional	Lithuania	*n* = 16,199 (8,131 girls, 8,068 boys): 5,775 in 1992, 5,325 in 2002, 5,099 in 2012; aged 11–18 years	Flamingo balance (*n*/min), Sit-and-reach (cm), Standing broad jump (cm), Sit-ups (n/30s), bent-arm hang (s), 10 × 5 m speed shuttle run (s), 20 m shuttle run (*n* stages); Eurofit test battery	The study has shown a loss of flexibility, leg muscle power, upper body strength, and cardiorespiratory fitness between 1992 and 2012, although there was an improvement in abdominal muscle strength in girls, agility in boys, and balance in both genders during the same period. Negative trends in aspects of fitness seen between 1992 and 2002 have not slowed down between 2002 and 2012. Positive trends in agility and abdominal muscle strength were seen before 2002 have regressed or were reversed between 2002 and 2012, while balance continued to improve at an increased pace	Strong
Costa et al. (19)	Cross-sectional	Portugal	*n* = 1,819: 881 boys, 938 girls; aged 10–11 years	Horizontal jump (without preparatory running) (cm), 40 m sprint (s) from AAHPERD test batteries; Curl-up (*n*), Sit-and-reach (cm) from the FITNESSGRAM test batteries	Children from the 1993 cohort were less flexible than those from 2013. Boys in 2013 were faster than their 1993 counterparts. A similar trend was found for girls but without statistical meaning. The curl-up assessment, showed a similar pattern in both genders, with better scores reached in the most recent quinquennial. Horizontal jump performance also showed slight improvements throughout those years but without reaching the significance of cut-of value	Moderate
Ao et al. (34)	Cross-sectional	China (Han ethnicity)	*n* = 136,539: 34,238 (in 1985); 11,664 (in 1991); 17,485 (in 1995); 18,057 (in 2000); 19,254 (in 2005); 17,962 (in 2010); and 17,906 (in 2014); aged 12 years	50 m run (s), Standing broad jump (cm), 10 × 50 m run (s), Pull-ups (*n*/min), Sit-ups (*n*/min); A battery test from the Chinese National Measurement Standards on People's Physical Fitness for young children	There was a general decline in physical fitness in both urban and rural children after 2000. Some components have upward trends: running speed in boys and girls in urban areas, cardiorespiratory fitness in boys and girls in both urban and rural areas	Strong
Huotari et al. (35)	Cross-sectional	Finland	*n* = 3,736: 2,390 students (1,207 boys, 1,183 girls) in 2003, 1,346 students (683 boys and 663 girls) in 2010; aged 15–16 years	Figure 8 (*n*/min), Lateral jumping (*n*/15 s), Motor coordination track (s)	Results demonstrate that scores of the coordination track decreased slightly in both gender groups between 2003 and 2010. Scores for the Figure 8 test increased slightly among girls but not in the boys' group between 2003 and 2010. There were no significant changes in the lateral jumping test scores in either gender group between 2003 and 2010	Moderate
Colley et al. (23)	Cross-sectional	Canada	*n* = 6,284: 2,081 (2007–2009), 2,133 (2009–2011), 2,070 (2016–2017); aged 6–19 in 10–year period	mCAFT step, Handgrip strength (kg), Sit-and-reach (cm); CSEP-PATH Manual	The fitness measures have changed across the three cycles (2007–2009, 2009–2011, 2016–2017), by age group and sex. Statistically significant differences between cycles are noted. Decreases in cardiorespiratory fitness were observed for 8- to 14-year-old boys. Grip strength decreased in 11- to 19-year-old boys. Flexibility was stable across time with a slight improvement observed in 6- to 10-year-old girls	Strong
Dong et al. (36)	Cross-sectional	China	*n* = 1,494.485: 409.836 (in 1985), 204,763 (in 1995), 216,073 (in 2000), 234,289 (in 2005), 215,223 (in 2010), 214,301 (in 2014); aged 7–18 years	Forced vital capacity (ml), Standing long jump (cm), Sit-and-reach (cm), Oblique body pull ups (*n*) and pull ups (*n*) for boys; sit ups (*n*/min) for girls), 50 m dash (s), 8 × 50 m shuttle runs (s), 1,000 m run (s) for boys, 800 m run (s) for girls	The mean levels of the six core items of the physical fitness indicator shifted substantially over this period. The total normal physical fitness indicator increased between 1985 and 1995, reached its peak in 1995, and then decreased in 2014, with overall physical fitness 167% lower between 1995 and 2014. Except for sit-and-reach, the other components of fitness (forced vital capacity, standing long jump, body muscle strength, 50 m dash, endurance running) significantly declined over time from 1995–2014, particularly forced vital capacity and endurance running	Strong
Bi et al. (37)	Cross-sectional	China	*n* = 49,357 participants: 14,548 (in 1985), 7,198 (in 1995), 10,255 (in 2005), 17,356 (in 2014); aged 7–18 years	Forced vital capacity (ml), Standing long jump (cm), Sit-and-reach (cm), Oblique body pull-ups (*n*) and pull-ups (*n*) for boys; sit ups (n/min) for girls, 50-m dash (s), 8 × 50 m shuttle runs (s), 1,000 m run (s) for boys, 800 m run (s) for girls; CNSSCH guidelines	Comprising the six core physical fitness items (forced vital capacity, standing long jump, sit-and-reach, body muscle strength, 50-m dash, endurance running), the physical fitness indicator increased in 1995 and then fell sharply in 2005 and continued to decrease in 2014, taking the 1985 dataset as reference. The physical fitness indicator of all age groups reached a peak in 1995, followed in descending order by 1985, 2005, and 2014	Strong
Johansson et al. (15)	Cross-sectional	Sweden	*n* = 705 children: 356 girls, 349 boys; aged 8–20 years.	Maximal oxygen uptake VO2 max (Astrand-Rhyming submaximal bicycle test)	There was a statistically significant negative time trend for cardiorespiratory fitness in both sexes. Absolute VO2 max (L/min) decreased in girls and in boys per year. Relative VO2 max (mL/kg/min) decreased in girls and in boys per year	Moderate

### Strength

Strength was mostly tested topographically (arm and shoulder belt strength in 10 papers for boys and eight for girls; lower limb strength in 10 papers for boys and 11 for girls; abdomen strength in five papers for boys and nine for girls). With the exception of one study, which applied a test assessing the strength of the entire body, eight tests were used to evaluate arm and shoulder strength: handgrip strength (kg), bent-arm hang (s), arm pull (kg), bench-press (n): all four for both boys and girls; pull-ups (n), oblique body pull-ups (n), pull-ups (n/min): three for boys only, and flexed-arm hang (s): only for girls. Three tests were used to evaluate the strength of the lower limbs in both sexes: standing long jump (cm), vertical jump (cm), and leg lifts. The sit-ups test was used for both sexes to evaluate the strength of the abdomen. In one study, a two-hand lift (N) test, which assessed the strength of the entire body, was used for boys and girls.

For the strength of the arms and shoulder belt, a declining trend was found in nine studies for boys and seven for girls. In three Chinese studies (34, 36, 37), a growth trend from 1985 to 1995 was initially found for all cohorts of boys, followed by a trend of decline until 2014. In the additional six studies for boys (3, 22, 23, 26, 27, 33) and seven for girls (3, 3, 23, 26, 27, 30, 31, 33), a steady decline was observed between 1969 and 2017. For a cohort of Chinese 12-year-olds, the results of upper-body strength showed an increase until 2005, followed by a decline (34). Significant changes were not shown in the cohort of Canadians aged 15 to 19 (23), in Finnish adolescents aged 13 to 16 (22), in boys in Belgium (27), and in Spanish boys and girls on a static strength test (3). Finally, a growth in strength was noted in Mozambique children and adolescents between 1992 and 2012 (30), in Canadian girls aged between 11 and 14 (23), and in Polish girls aged 15.5 years on an absolute strength test (31).

For the explosive strength of the lower extremities, a declining trend was found in six studies for boys and eight for girls. Three Chinese studies for boys and girls (34, 36, 37) found an initial growth trend from 1985 to 1995 (it peaked in 1995), followed by a downward trend until 2014. In three studies for boys and girls (3, 28, 33) and two girls only (27, 31), a steady decline was noted between 1969 and 2013. No significant changes for explosive strength were noted in the study by Huotari et al. (22) of Finnish boys, the study by Westerstahl et al. (26) for Swedish girls, and in the study with Portuguese adolescents of both sexes (19). Furthermore, no significant changes were noted for the repetitive strength of the subjects of both sexes in Belgium (27). Progress was recorded for a cohort of Swedish adolescents aged 16.4 between 1974 and 1995 (26), which is consistent with the results of studies in China in which a decline in strength occurs after that period. The growth trend was recorded for a cohort of Finnish adolescents aged 13–16 between 1976 and 2001 (22).

The trend of abdomen strength decline in both sexes was found in Swedish adolescents between 1974 and 1995 (26), and in Polish girls (31) between 2006 and 2013. A cohort of Portuguese school-aged boys from 1993 to 2008 and girls from 1993 to 2003 found a trend of decline, followed by a growth trend until 2013 (19). Studies in China have noted a trend of decline in repetitive strength in girls from 1985 to 1995, followed by a growth trend (34, 36, 37). The growth of repetitive strength in both sexes was recorded in New Zealand from 1991 to 2003 (28) and in Finland between 1996 and 2002 (22). In contrast, between 1992 and 2012, only Lithuanian girls showed an increase, while there were no significant changes among the boys (33).

Westerstahl et al. (26) examined complete body strength and found progress for both sexes of a cohort of Swedish adolescents aged 16.4 years, for the period between 1974 and 1995, which is a similar result to those in Chinese studies indicating fitness growth also up to 1995 and reaching a peak in that period (34, 36, 37).

### Endurance

Endurance was assessed in boys within 14 research studies and in girls within 15 studies. Eleven tests were used for both sexes: 10 × 50 m run (s), 1-mile run-walk (s), 20 m shuttle run (*n* stages), 3,000 m running (s), 550 m run (s), 8 × 50 m shuttle run (s), Bruce protocol treadmill test, Astrand-Rhyming submaximal bicycle test, cycle test with progressively increasing workload, mCAFT step, run-walk (m/9 min). Two tests were used only for boys [1,000 m run (s) and 2,000 m run (s)] and two for girls only [1,500 m run (s) and 800 m running (s)]. Two studies have applied a vital capacity (ml) test.

The constant trend of decline in boys' and girls' endurance is indicated by the results of eight studies covering the period between 1969 and 2017 [(Huotari et al., 2009), (15, 23, 26, 28, 30, 33)]. Studies in China did not show significant changes in results for both sexes from 1985 to 1995 followed by a period of continuous decline (36, 37). Dyrstad et al. (17) pointed to the growth of endurance of Norwegian adolescents from 1969 to 1989 and for girls from 1980 to 2000, and its decline between 1990 and 2009 for boys and between 2000 and 2009 for girls. A study in Canada (23) for an older cohort of boys and girls (15–19 years) showed no significant changes between 2007 and 2017, as well as in Danish adolescents of both sexes between 1983 and 2003 (29). Some studies pointed to the growth in the endurance of some cohorts, such as Chinese 12-year-olds (34) of both sexes (Han ethnicity) between 1985 and 2014, Spanish adolescents of both sexes aged 12.5 to 17.5 years between 2001 and 2007. (3), and girls in Poland (15.5 years) from 2006 to 2013 (31).

Two studies have shown a trend for forced vital capacity (ml) that shows functional ability, as people with high lung capacity are predisposed to endurance-type sports. The results for this parameter were calculated together for both sexes for children and adolescents aged 7–18 years and show inconsistent results. For the entire population of China, forced vital capacity decreased from 1985 to 2005, after which it increased until 2014 (36), while for residents in Xinjiang (China) it increased until 1995 (reached a peak in 1995), then declines until 2005 after which it remains at a similar level until 2014 (37).

### Flexibility

Flexibility was tested within nine research studies for both sexes. For boys, a sit-and-reach test was used, and a sit-and-reach test and standing trunk flexion were used for girls. Tests in Belgium (27), Lithuania (33), Portugal (19), and testing of girls in Poland (31) show a decrease in the flexibility between 1969 and 2013. In China, from 1985 to 1995, progress was seen (reaching a peak in 1995), after which the results decreased until 2014 for both sexes (37). The opposite trend is cited by another Chinese study showing a decline in the flexibility of boys and girls until 1995, followed by an increase to the year 2000 and retention at similar values until 2010, and finally a slight decline until 2014 (36). Colley et al. (23) found a decrease in flexibility for the period between 2007 and 2009 and then an increase until 2017 in a sample of Canadian boys aged 15–19, while for boys between 11 and 14 years they found no significant change in the results of the flexibility test. The same authors demonstrate girls' progress of flexibility for both ages. The growth of flexibility for both sexes is reasonable in the cohort of school children (10–14 years) of New Zealand (28) for the period between 1991 and 2003 and children (8–15 years) in Mozambique (30) for the period between 1992 and 2012.

### Agility

Agility was tested within six studies for boys and within seven studies for girls. Three tests for both sexes were used for assessment: a 10 × 5 m shuttle run (s), a 4 × 10 m shuttle run (s), and a 4 × 9 m run (s). In both sexes, agility declines in Mozambique (30), and New Zealand (28), moreover, for girls in Belgium (27) and Poland (31). The growth of agility for both sexes was found in Spain (3) and Finland (22) and only for boys in Lithuania (33), while the girls showed no significant changes. In Belgium, there is a trend of increasing results for boys between 1969 and 2005, but there were no significant changes between parents and their children (27).

### Speed

Speed testing was performed within six studies. For the evaluation of both sexes, the following tests were used: 50 m sprint (s), 40 m sprint (s), and the speed of movement frequency was measured by the Plate tapping test (*n*/20 s). Tests show a negative trend in the speed of boys and girls in the cohort of Flemish subjects in Belgium (27) and in girls in Poland (31). In a study by Ao et al. (34), Chinese girls' (Han ethnicity) speed decreased from 1985 to 1991 and increased thereafter but only in rural areas, while in boys a constant decrease was recorded. In the Chinese province of Xinjiang, the speed decreased for both sexes from 1985 to 1995, then increased until 2005, after which it remained stable (37). In contrast, the results for the whole of China for both sexes (36) show an increase in speed until 1995, then a decrease until 2005 and retention at a similar level until 2010, followed by a slight increase in 2014. The increase in speed was also visible in urban areas of China (Han ethnicity) for 12-year-olds of both sexes (34), and Portugal (19).

### Balance

Balance was tested for both sexes as part of two research studies with the Flamingo balance (*n*/min) test. Balance tests show that in the cohort of Flemish subjects in Belgium (12–18 years) there are no significant changes when comparing the results of parents and their children later when they reached the same age; however, for the entire female population there is a decrease in balance (27). In Lithuania, greater improvement in balance scores was found in the previous decade and still more in girls 11–18 (33).

### Coordination

Only one study shows coordination results for both sexes using three tests: motor coordination track (s), lateral jumping (*n*/15 s), and Figure 8 (*n*/min). The only study that examined coordination was conducted by Huotari et al. (35) for a cohort of Finnish adolescents (15–16 years) in the period between 2003 and 2010. The motor coordination track (s) test shows a decrease during the tracked period for both sexes. Lateral jumping test (*n*/15 s) which evaluates dynamic balance, quickness, and the explosive strength of lower limbs with coordination indicates that there are no significant changes for both sexes, while the Figure 8 test (*n*/min) test, which evaluates object control skills with coordination, shows that there are no significant changes in boys, while girls have made progress in results.

## Discussion

This study provides a comprehensive overview of longitudinal changes in the physical fitness of children and adolescents and thereby indicates relevant knowledge to develop appropriate public health strategies. The articles that were included in the qualitative synthesis describe the temporal trends for seven physical fitness attributes. Overall, a declining trend was found for one or all three topological areas of strength (9 of 10 studies for boys and in 8 in 11 for girls), endurance (9 in 14 studies for boys and in 8 in 15 for girls), flexibilities (4 in 9 studies), agilities (4 in 6 studies for boys and 4 in 7 for girls), and speed (2 in 6 studied).

Based on the analysis of all three topological areas of strength (arm and shoulder belt strength, lower limb strength, abdomen strength), it can be noted that most studies indicate a declining trend, which is not surprising given the changing lifestyle of children and adolescents, physical inactivity, and increasing screen-time around the world in the previous three decades (38). However, in some studies, in certain cohorts a trend of increase in strength has been noticed whereas in other cohorts a decrease in strength was not evident (19, 22, 23). It should be emphasized that these are studies with children and adolescents in which (according to the decisions of the governments in these countries) special programmes of additional exercise were applied. Possibly, even in these cohorts there would have been a declining trend in strength levels if there had been only the regular school curriculum, without additional exercise. In several studies, the handgrip strength test shows a growth trend in strength for girls (23, 31, 36). These results are in line with previous research, since absolute strength is proportional to the size of the muscle cross-sectional area (39) and body height, and which increased over the last 40 years (11, 12).

Noteworthy is the fact that the majority of studies point to a trend of declining levels of endurance, which can be explained by the same causes as for the trend of declining strength. A small number of cohorts in certain studies show that there is no decline in endurance levels or even a growth trend. However, these are cohorts for which intervention programmes mirror the longitudinal effects (23, 31) or studies covering insufficient periods (3), or studies with non-representative populations (29, 34). In summary, the results of these studies must be interpreted with caution, when drawing conclusions about longitudinal trends.

Notably, research conducted in Europe has recorded a trend of declining levels of flexibility. In other parts of the world, growth trends have been reported, for example, in Canada (23), where additional exercise programmes have been conducted. However, the reasons for the growth trend in New Zealand (28) and Mozambique (30) have not yet been determined.

Given the diversity of agility trends in the available studies, it is not possible to discuss a specific direction of movement for this motor ability in children and adolescents. The speed trends are very difficult to interpret because results indicate differences. In particular, the three Chinese studies are quite contradictory. The trend of increasing speed in Portugal (19) probably results from the government's policy on additional exercise, although it is known that speed is highly genetically determined.

Two studies examining the balance are not enough to define the trajectory of the global trend. The results of studies conducted in Belgium (27) and Lithuania (33) are contradictory at first glance, but they are focused on different time periods, and it is possible that in identical periods differences would not exist.

Only one study that have examined coordination (35) does not provide a possibility to discuss global trends.

Generally, the decline of physical fitness of children and adolescents around the world is caused by various factors. In recent years, several studies showed that weight gain is related to physical fitness (14, 15, 31). As body weight increased, so did BMI, which influenced this trend in China (37), Sweden (26), and New Zealand (28). In addition to these two components, the increase in the thickness of the skin folds caused a decline in physical fitness in Belgium (27). Two Chinese studies showed that the decline in physical fitness was caused by changing lifestyles, characterized by higher media and fast food consumption (34, 36). Morales-Demori et al. (32) linked the trend of declining endurance to a sedentary lifestyle, and Venckunas et al. (33) with additional risk factors of smoking, alcohol consumption, non-active lifestyle and long-term television viewing.

This study also has certain limitations. One of the most significant is the insufficient differentiation of the samples by age (e.g., 10–12; 12–14; 14–16; 16–18) which would show the most accurate data. However, the results for the whole population are mostly combined. Also, the authors themselves are self-critical and reported the following limitations in their works: some studies combined results for both sexes; field tests although used worldwide with this age, carry the possibility of individual errors of measurement performers; when testing aerobic fitness, the results will have real values only if all subjects are highly motivated; in some studies urban i rural area are divided, but did not take into account the urbanization of certain rural areas, which likely cause a certain contradiction; results of non-representative samples of some studies can't be generalized for the whole population. Finally, it should be noted, that causes and determinants of physical fitness trends have not been fully identified by researchers. However, weight gain and higher BMI levels are mainly the cause of the declining trend in physical fitness, which facilitates the need to monitor several health-related factors (e.g., food intake) among several settings of the living environment.

Studies from Finland (22), Canada (23), and Portugal (19) show that changing policies, including the development and implementation of health-enhancing programmes, can reduce the negative impact of a sedentary lifestyle, which have taken precedence in our population.

Finally, consistent conclusions about the development of strength and endurance were drawn, because these dimensions were assessed with the largest number of tests, while the other motor skills (flexibility, agility, speed, balance, coordination) were less frequently and insufficiently tested to draw a meaningful conclusion. We encourage researchers to develop a comprehensive and easily applicable test battery enabling the assessment of all motor skills. Doing so would clearly enhance the evidence regarding the longitudinal trends of physical fitness among children and adolescents around the world.

## Data Availability Statement

The datasets presented in this study can be found in online repositories. The names of the repository/repositories and accession number(s) can be found in the article/supplementary material.

## Ethics Statement

Ethical review and approval was not required for the study on human participants in accordance with the local legislation and institutional requirements. Written informed consent to participate in this study was provided by the participants' legal guardian/next of kin.

## Author Contributions

BM wrote the manuscript, collected the data, and performed analyses. JG wrote the manuscript, overviewed previous studies, and discussed the results. AM performed analyses. MP discussed the results. YD revised manuscript. DS revised manuscript. SP discussed the results and revised manuscript. All authors contributed to the article and approved the submitted version.

## Conflict of Interest

The authors declare that the research was conducted in the absence of any commercial or financial relationships that could be construed as a potential conflict of interest. The reviewer AB declared a past co-authorship with the authors YD and DS.
